# Supplementary material for: The role of customer experience in the effect of online flow state on customer loyalty
Source: PLoS One. 2021 Jul 15;16(7):e0254685. doi: 10.1371/journal.pone.0254685 (PMC8282012; doi:10.1371/journal.pone.0254685)
Supplement: Attachment — Submitted filename: Response to Reviewers.docx [file pone.0254685.s002.docx]

1. Conformance to PLOS ONE’s style requirements

The manuscript has been revised to conform to the PLOS ONE’s style requirements. The changes have been highlighted in the manuscript with track changes open in the Word document.

2. Text overlap with 4 sources

The part of the text related with the mentioned 4 sources has been rephrased and the sources has been mentioned in the text.

3. Availability of the Research Dataset

We have uploaded the research dataset for your information.

4. Missing reference to Table 3 within the text

Additional sentence is put to reference Table 3.

**Reviewer Comments:**

Reviewer #2:

Significance of the study

New text has been added to the Introduction and Conceptual background parts to underline the significance of the study.

Reviewer #3:

1. Typing errors

The whole manuscript has been revised again to correct the typing errors including but not limited to the mentioned ones. The corrections have been highlighted with track changes on in the Word document.

2-3 Sampling and descriptive statistics

The following paragraph has been added to 4.1 Measures and Sampling part which includes answers to the raised questions.

The questionnaire was distributed to more than 700 individuals residing in Turkey via an online from. The survey was conducted in Turkish, among university students residing in 7 biggest cities in all geographical regions in Turkey, namely; İstanbul, İzmir, Antalya, Samsun, Ankara, Erzurum, Diyarbakır. The online questionnaire was accepted only from those adults with their explicit consent and who had previously purchased online in the past 12 months. 538 valid questionnaires from individuals were collected. 334 of the respondents were female and the remaining 204 respondents were male.

4-5-6-7-8 Authors Comments to the criticisms on Validity

The method of the article is very robust and the determination of validity is reported correctly. In a theoretically determined model, construct validity refers to convergence of observed variables that are connected to the same latent variable (convergent validity) and dissociation of observed variables from other observed variables that are connected to other latent variables (discriminant validity). The construct validity indicates that the observed variables do not measure any latent variable other than they connected in the conceptual model. But in this case, it would not be correct to say that the validity of the construct is fully realized without confirming the reliability of the scale (Gerbing & Anderson, 1988).

Convergent validity indicates that the correlations between questions constituting a construct are high. In structural equation modeling method, it is necessary to look at the results of confirmatory factor analysis to determine the convergent validity of the scales used to measure the dimensions constituting the conceptual model of the research. The measurement model part of structural equation models corresponds to confirmatory factor analysis (Confirmatory Factor Analysis - CFA). Therefore, if the measurement model fit indices are low, there is no need to test the structural model (See also Figure 1. Demarcation between Measurement Model and Structural Model). Because the scales used to measure the dimensions that make up the conceptual model will not be validated. Therefore, if the measurement model is insufficient, the fit indices of the structural model will be low. The t test results of all the coefficients in the measurement model should indicate that the coefficient values are different from zero. The standard value of each coefficient in the measurement model is the factor loadings of the confirmatory factor analysis. Each factor load should be higher than 0.50. Otherwise, the fit indices of the general model will be adversely affected. The fact that the factor loads are above 0,5 is evidence of convergent validity. If the critical rate value of a question in CFA results is greater than 2 as an absolute value this means that this item is loaded to the factor it is connected.

Discriminant validity is the measure of the level at which a structure in a measurement model differs from other structures. It is an indicator of a low correlation between the questions that form a construct and other questions that form another construct. To find the discriminant validity for each dimension, we first need to calculate the Average Variance Extracted (AVE) value for each dimension. The acceptable AVE value must be greater than 0.50 or 0.50. However, as noted in the previous section, this value confirms convergent validity when examined alone (Fornell & Larcker, 1981). In order to determine discriminant validity, it is also desirable that the values of the AVE for each construct in the data set are larger than the correlation coefficients of that construct with the other constructs. In this case, it can be determined that the scales used have discriminant validity for each dimension. AVE value alone does not indicate discriminant validity but the square root of the AVE value of each construct is larger than the inter-dimensional correlation value it can be said that there is discriminant validity (Fornell & Larcker, 1981).

9. Bibliography

The extant literature has been revised extensively including but not limited to the recent literature.

10. Reference names

References section has been revised thoroughly to correct any mistakes.

**Reviewer #4**

Typing errors have been corrected. The corrections are highlighted with track changes open.

The second paragraph in the introduction section has been removed and the third paragraph has been revised.

New sentences were added to discuss the Covid-19 scenario also in the introduction.

Note to the reviewer: No pre-test was carried out in the research.
